# Supplementary material for: Virulence network of interacting domains of influenza a and mouse proteins
Source: Front Bioinform. 2023 Feb 17;3:1123993. doi: 10.3389/fbinf.2023.1123993 (PMC9982101; doi:10.3389/fbinf.2023.1123993)
Supplement: Supplementary file 1 [file DataSheet1.PDF]

frontiers

# *Supplementary Material*

## SUPPLEMENTARY FIGURES

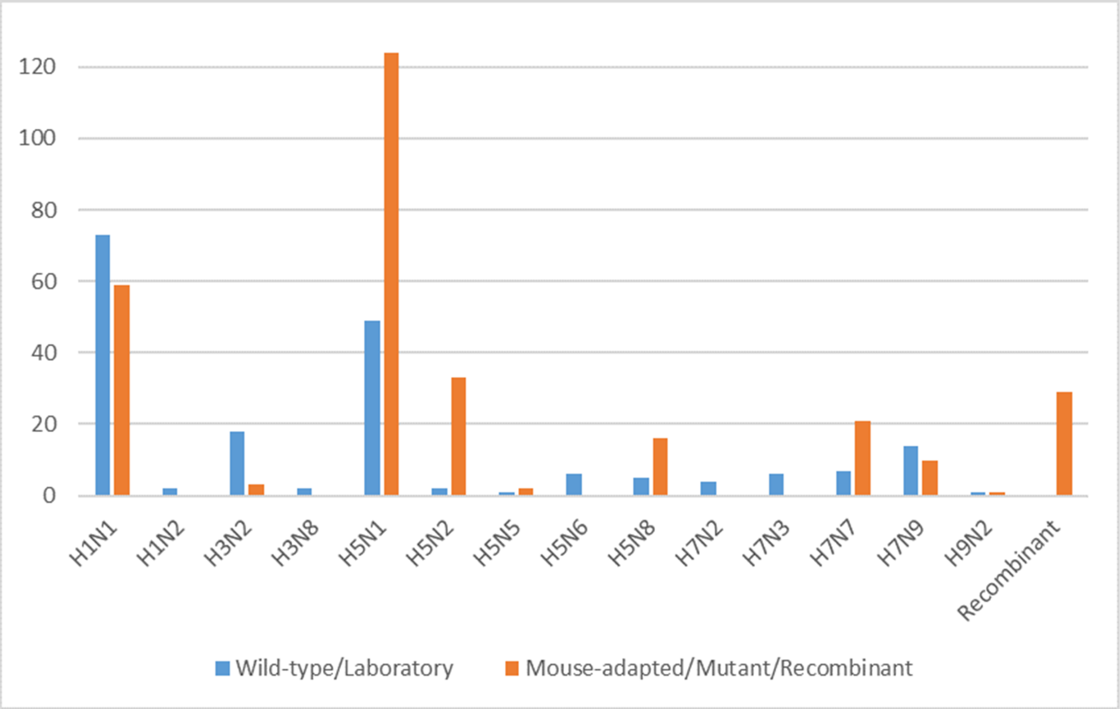


**Figure S1.** Initial dataset of 488 infection records - Proportion of wild-type/laboratory against mouse-adapted/mutant IAV strains. 'Recombinant' refers to IAV formed by the combination of protein segments retrieved from at least two different IAV subtypes. Infection records involving mouse-adapted, mutant and recombinant IAV strains (as represented by the orange bars) were first omitted, reducing the number of infection records to 190.

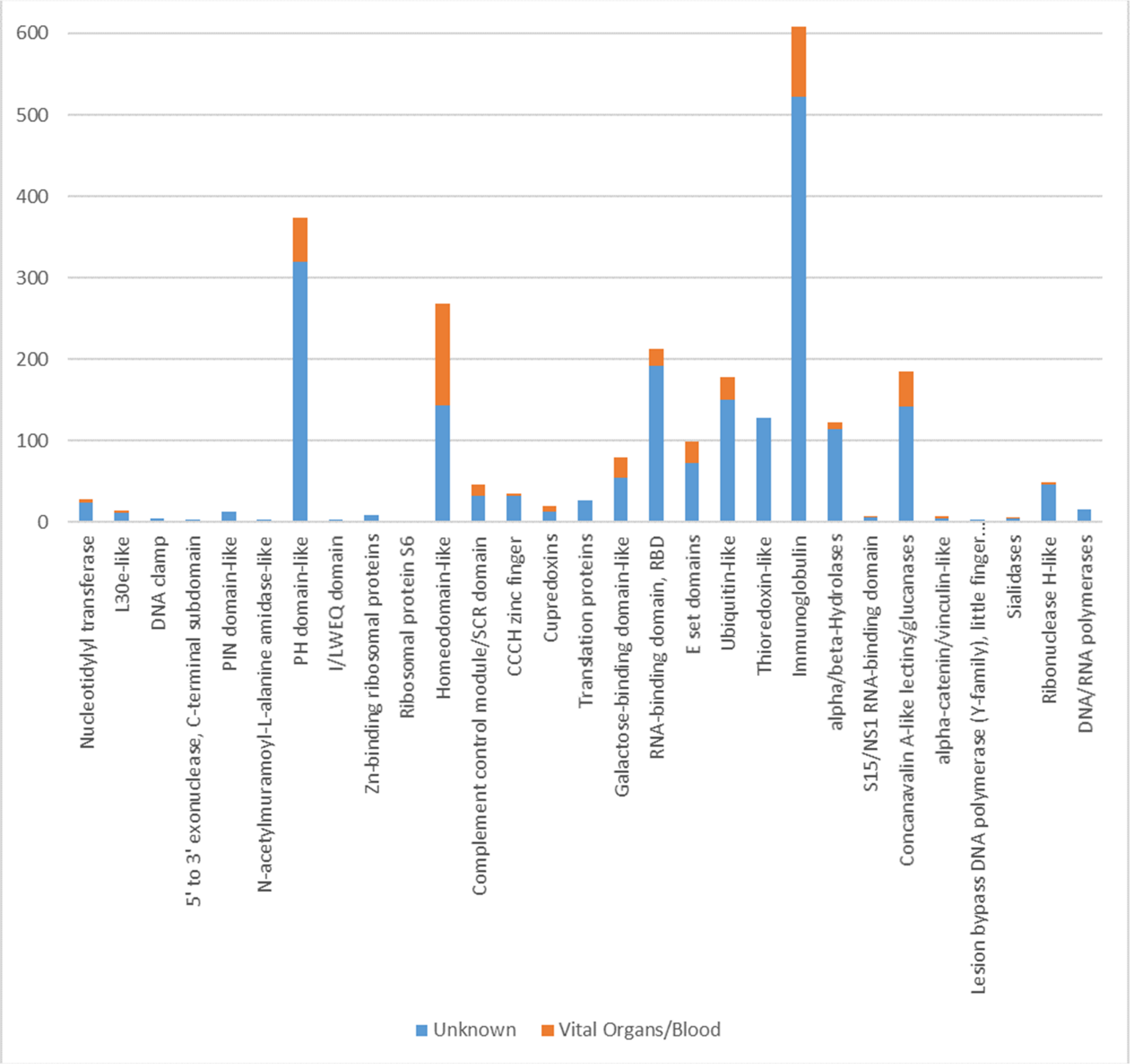


**Figure S2.** Mouse protein tabulation, grouped according to the 29 interacting SCOP superfamilies. ‘Vital Organs/Blood’ refers to the aggregation of mouse proteins that can be found in a mouse’s lungs, brain, liver, kidney, spleen, heart and blood. ‘Unknown’ refers to the remaining mouse proteins, found in all other parts of a mouse’s body.
